# Attitudes, and practices toward allergic rhinitis: a comparative cross-sectional study of patients and non-patients in China

**DOI:** 10.3389/fmed.2026.1807065

**Published:** 2026-06-08

**Authors:** Y. X. Y Ding, Su Lan, Shipeng Zhang, Yanjie Jiang, Xinrong Li, Qinxiu Zhang, Fu Li

**Affiliations:** 1 Hospital of Chengdu University of Traditional Chinese Medicine, Chengdu, China; 2 Chengdu University of Traditional Chinese Medicine, Chengdu, China; 3 Nanjing University of Chinese Medicine, Nanjing, China

**Keywords:** adult, AR, KAP, OR, risk

## Abstract

**Background:**

Allergic rhinitis (AR) is a common immunoglobulin E-mediated inflammatory disease of the nasal mucosa, with a globally rising prevalence. Patient knowledge, attitudes, and practices (KAP) significantly influence disease management, yet systematic KAP assessments comparing AR patients and non-AR participants remain limited.

**Objective:**

This study aimed to evaluate and compare the KAP levels related to AR between patients diagnosed with AR and non-AR individuals, and to identify factors associated with knowledge levels.

**Methods:**

A cross-sectional web-based survey was conducted among patients attending the otolaryngology department of a tertiary hospital in Chengdu, China, from September to December 2022. A self-designed KAP questionnaire covering demographic characteristics, AR-related knowledge (20 items), attitudes (3 items), and preventive practices was administered. Knowledge scores were categorized into “adequate” (≥ 12) and “inadequate” (< 12). Logistic regression analyses were performed to identify factors associated with knowledge levels.

**Results:**

Among 933 valid respondents, 462 (49.51%) were diagnosed with AR. AR patients had significantly higher overall knowledge scores than non-AR participants (mean 14.0 vs. 10.6, *p* < 0.001). Both groups showed knowledge gaps regarding environmental and lifestyle risk factors such as housing renovation, humidity, probiotic consumption, smoking exposure during pregnancy, and delivery mode. In terms of attitudes, AR patients expressed stronger desire for curative drugs, while non-AR participants were more interested in understanding disease etiology. A majority of AR patients (64.72%) lacked confidence in current treatments. AR patients also reported stronger intentions to adopt preventive practices (*p* < 0.05). Logistic regression identified AR diagnosis (OR = 3.69, 95% CI: 2.80–4.85) and female gender (OR = 1.54, 95% CI: 1.17–2.04) as significant predictors of adequate knowledge.

**Conclusion:**

AR patients possess better AR-related knowledge and stronger behavioral intentions than non-AR individuals, yet critical knowledge gaps and low treatment confidence persist. Targeted health education should address specific knowledge deficiencies and integrate psychological support to improve patient trust and self-management.

## Introduction

Allergic Rhinitis (AR) is an IgE-mediated, non-infectious inflammatory disorder of the nasal mucosa, clinically characterized by paroxysmal sneezing, watery rhinorrhea, nasal pruritus, and nasal congestion, which significantly impairs patients’ quality of life (1). In recent years, driven by environmental and lifestyle changes, the global prevalence of AR has been steadily increasing, affecting approximately 10%–30% of the population, with even higher rates observed in some developed regions. AR has thus emerged as a major public health concern (2, 3). Frequently diagnosed in children and young adults, AR often coexists with other atopic conditions such as asthma and atopic dermatitis, forming a comorbid pattern consistent with the “one airway, one disease” concept. This pattern contributes to a substantial disease burden and imposes considerable socioeconomic costs (4, 5).

However, despite the relative maturity of diagnostic and therapeutic strategies for AR—such as pharmacotherapy and allergen-specific immunotherapy recommended by the ARIA guidelines—clinical management still faces challenges including underdiagnosis and poor treatment adherence (6). Many patients possess limited disease awareness, frequently mistaking symptoms for the common cold, or discontinuing treatment due to concerns about the safety of intranasal corticosteroids or dissatisfaction with their speed of onset, leading to suboptimal disease control. These difficulties highlight the limitations of a purely biomedical model and underscore the critical role of patients’ cognition, attitudes, and behaviors in disease management (7, 8).

The Knowledge, Attitude, and Practice (KAP) theory is widely employed to explain health-related behaviors and has been extensively applied in medical clinical research to assess healthcare providers’ and patients’ understanding and behaviors regarding various health conditions (9, 10). Rooted in health education and social psychology, the KAP theoretical framework operates on the premise that “knowledge influences attitude, and attitude guides behavior.” It utilizes structured questionnaires to evaluate target populations across three dimensions: disease-specific knowledge, beliefs and attitudes, and actual practices (9). Numerous KAP studies have historically aided clinicians in understanding the current landscape of clinical diseases. For instance, a recent KAP study on esophageal cancer revealed that participants demonstrated moderate knowledge, neutral attitudes, and suboptimal practices, with the findings offering targeted clinical recommendations for health education (11). Similarly, a KAP survey on pediatric ophthalmic surgery conducted by Dr. Li in China suggested that better parental knowledge of pediatric ophthalmology may lead to improved practices, ultimately contributing to better treatment outcomes for pediatric ophthalmic surgery patients (12).

Knowledge, Attitude, and Practice survey research not only reflects clinical issues but also provides valuable reference points to inform physicians’ treatment decisions. Therefore, conducting a KAP study on AR holds significant theoretical and practical importance. In the context of AR, KAP surveys can help systematically identify cognitive misconceptions, attitudinal biases, and behavioral patterns among patients and caregivers. This, in turn, can provide an evidence-based foundation for developing targeted health education programs, enhancing treatment adherence, and improving quality of life.

## Materials and methods

### Study design

This study was a web-based cross-sectional survey designed to assess the KAP levels of patients attending the Department of Otolaryngology at the Affiliated Hospital of Chengdu University of Traditional Chinese Medicine, and to analyze related influencing factors. The survey was conducted using an anonymous self-administered electronic questionnaire, with data collected between September and December 2022. The study protocol was approved by the Ethics Committee of the Affiliated Hospital of Chengdu University of Traditional Chinese Medicine (Approval No.: 2021KL-015). All participants read an electronic informed consent form prior to completing the questionnaire. Participation was voluntary, and participants consented to the use of their data for scientific research.

### Study participants

The study participants were patients attending the Department of Otolaryngology at the Affiliated Hospital of Chengdu University of Traditional Chinese Medicine, including minors whose questionnaires were completed by their parents. Based on clinical diagnosis, participants were categorized into AR and non-AR groups. (Note: This study was a cross-sectional observational study. The non-AR group serves solely as a baseline comparison group to assess differences in KAP levels between the two groups, rather than functioning as an experimental control).

**FIGURE 1:**
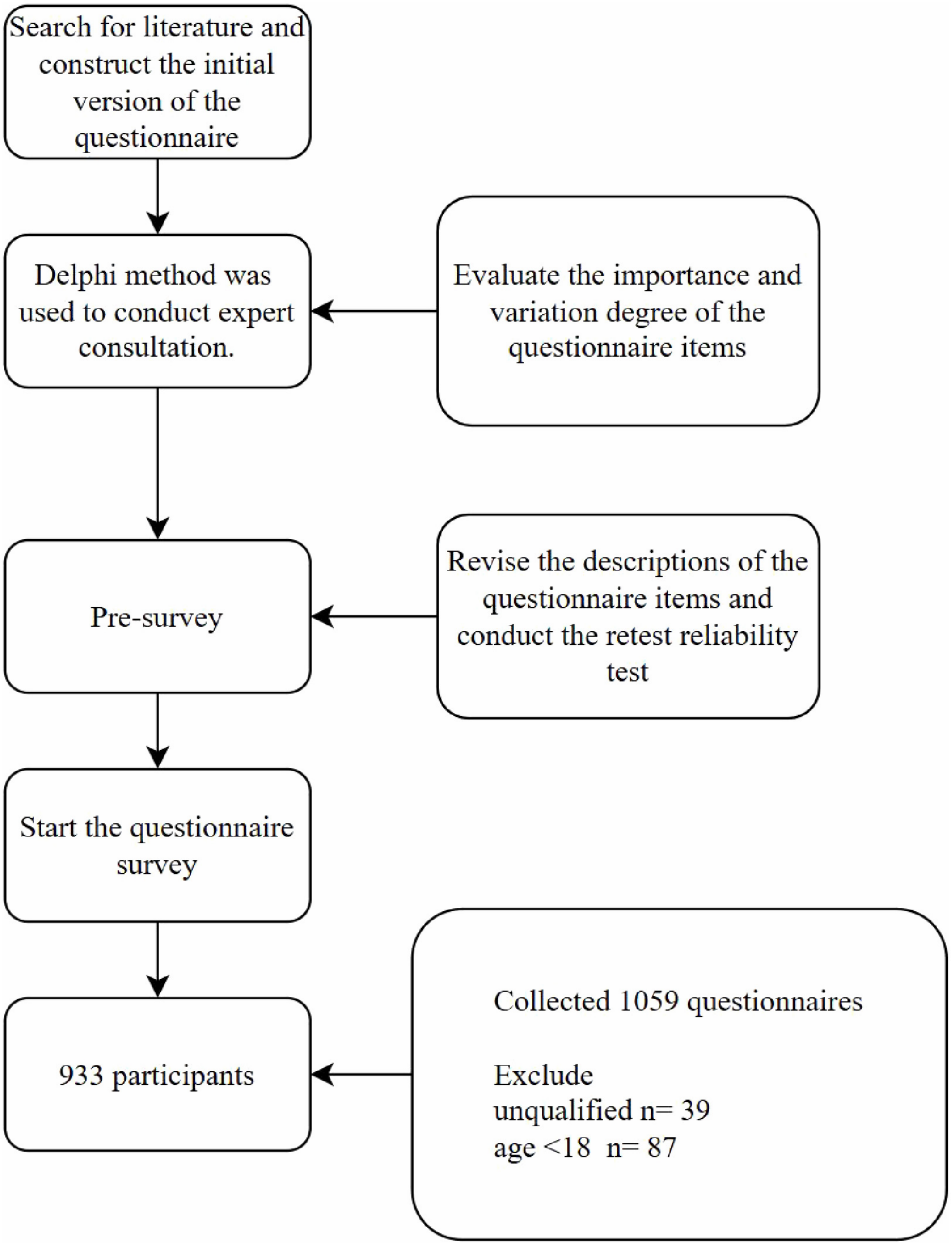
Flowchart of questionnaire design and participant recruitment process.

Using the convenience sampling method, patients who visited the hospital from September to December 2022 were invited to voluntarily scan the code and fill out the questionnaire. A total of 1059 questionnaires were initially collected. Following logical verification and completeness screening, 1020 valid questionnaires were included. To improve data accuracy, questionnaires from minors (aged under 18 years) were subsequently excluded, resulting in a final analytical sample of 933 participants (Figure 1). (The reasons for the subsequent exclusion were to ensure the homogeneity of the adult sample, as there are significant differences in cognitive abilities and disease perception between minors and adults).

### The diagnostic criteria for AR

The diagnosis of AR strictly follows the “Allergic Rhinitis and Its Impact on Asthma (ARIA) 2016 Revised Guidelines” (1). It requires that the typical clinical symptoms (sneezing, runny nose, nasal itching, nasal congestion) persist for at least 1 h daily, and either a positive SPT or a positive serum-specific IgE test result is obtained.

### Survey instrument

The “Allergic Rhinitis-Related Risk Factors KAP Questionnaire,” a self-designed instrument, was administered online via the Wenjuanxing platform. The questionnaire comprised four sections:

Sociodemographic Characteristics: Including age, gender, educational level, occupation, geographical location, marital status, and professional background.

Knowledge Assessment: This section consisted of 20 items covering risk factors (e.g., pollen, dust mites, smoking, air pollution, genetic factors, diet, mode of delivery) and preventive knowledge related to allergies. Responses were recorded as “Yes,” “No,” or “Unclear.” Correct answers were scored 1 point each, yielding a total possible score ranging from 0 to 20. Higher scores indicated a greater level of allergy-related knowledge.

Attitude Assessment: This section included 3 items evaluating disease perception, confidence in treatment, and empathy.

Practice Assessment: This section assessed behavioral intentions, such as wearing masks for prevention and lifestyle modifications.

The questionnaire was developed with reference to national and international guidelines and literature on allergic rhinitis. Its content validity was ensured through review by experts in otolaryngology and public health. The questionnaire underwent reliability and validity testing. Results indicated a Cronbach’s α coefficient of 0.78 and a KMO value of 0.82, demonstrating good internal consistency reliability and structural validity (Supplementary material).

### Statistical analysis

Data analysis was performed using SPSS software (version 26.0). Categorical variables were expressed as frequencies and percentages. Continuous variables were described using mean ± standard deviation or median. Inter-group comparisons were conducted using the Chi-square test or *t*-test. Based on the knowledge scores, a cut-off point of 12 was established. Participants scoring ≥ 12 were classified as the “relatively knowledgeable” group, while those scoring < 12 were classified as the “relatively knowledge-deficient” group. Logistic regression models were constructed to explore factors associated with knowledge scores, adjusting for potential confounders such as age, gender, educational background, and family history. A *p*-value of < 0.05 was considered statistically significant. To verify the robustness of the survey results, we conducted a sensitivity analysis, using the median score of 11 in the questionnaire as the grouping criterion to test the reliability of the main analysis results.

## Results

As shown in Table 1, a total of 933 participants were included in this survey study. Based on diagnosis by clinical otolaryngologists, the prevalence of AR was as high as 49.51%. According to AR status, participants were categorized into the AR group (*n* = 462) and the non-AR group (*n* = 471). No significant differences were observed between the two groups regarding age, height, weight, body mass index (BMI), gender, educational level, marital status, or geographical distribution. Notably, the Knowledge score in the AR group was significantly higher than that in the non-AR group, with the difference being statistically significant. In the non-AR group, more than half of the participants scored below 12 points, while nearly one-third of the participants in the AR group scored below 12 points.

**TABLE 1:** Patient demographics and baseline characteristics.

Allergic rhinitis				
Characteristic	No *N* = 471^1^	Yes *N* = 462^1^	Statistic	*p*-value
Age			t = 1.44	0.151^2^
Mean ± SD	37.8 ± 9.2	37.0 ± 8.9		
Height			t = 0.860	0.390^2^
Mean ± SD	161.9 ± 7.0	161.5 ± 7.3		
Weight			*t* = −0.832	0.406^2^
Mean ± SD	61.6 ± 9.6	62.2 ± 9.3		
BMI			*t* = −1.268	0.205^2^
Mean ± SD	23.6 ± 4.1	24.0 ± 4.1		
Sex			χ^2^ = 0.012	0.730^3^
Male	201 (42.7%)	190 (41.1%)		
Female	270 (57.3%)	272 (58.9%)		
Education			χ^2^ = 1.889	0.389^3^
Graduate student	125 (26.5%)	105 (22.7%)		
High school and below	228 (48.4%)	232 (50.2%)		
Undergraduate	118 (25.1%)	125 (27.1%)		
Marital status			χ^2^ = 0.160	0.689^3^
Married	366 (77.7%)	364 (78.8%)		
Unmarried	105 (22.3%)	98 (21.2%)		
Family with Allergic rhinitis	284 (60.3%)	275 (59.5%)	χ^2^ = 5.620	0.018^3^
Home location			χ^2^ = 0.680	0.410^3^
City	219 (46.50%)	254 (54.98%)		
Rural areas	252 (53.50%)	208 (45.02%)		
Knowledge - Total, Mean ± SD	10.6 ± 2.3	14.0 ± 3.9	*t* = −16.05	< 0.001^2^
Knowledge ≤ 11	308 (66.67%)	154 (33.70%)	χ^2^ = 95.89	< 0.001^3^

^1^n (%)

^2^Welch Two Sample *t*-test

^3^Pearson’s Chi-squared test

**FIGURE 2:**
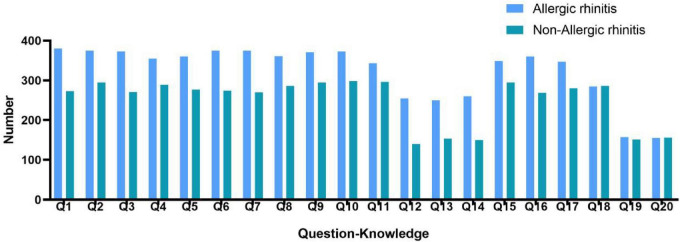
The distribution of knowledge between the AR group and the Non-AR group.

Figure 2 and Table 2 present the scoring patterns for each item in the knowledge section between the AR and non-AR groups. The results indicate that the number of correct respondents was consistently higher in the AR group than in the non-AR group for each item. With the exception of items Q18 to Q20, all other items showed statistically significant differences in the proportion of correct responses between the two groups, with a significantly higher number of correct responses in the AR group compared to the non-AR group (*P* < 0.05).

**TABLE 2:** A comparison of the scores of the two groups in the knowledge section.

AR					AR				
Characteristic	No	Yes	Cramer’s V	*p*-value	Characteristic	No	Yes	Cramer’s V	*p*-value
	*N* = 471	*N* = 462				*N* = 471	*N* = 462		
Q1, n (%)			0.265	< 0.001^1^	Q11, n (%)			0.1231	< 0.001
No	198 (42.04%)	82 (17.75%)			No	175 (37.15%)	119 (25.76%)		
Yes	273 (57.96%)	380 (82.25%)			Yes	296 (62.85%)	343 (74.24%)		
Q2, n (%)			0.206	< 0.001^1^	Q12, n (%)			0.2581	< 0.001
No	176 (37.37%)	87 (18.83%)			No	331 (70.28%)	207 (44.81%)		
Yes	295 (62.63%)	375 (81.17%)			Yes	140 (29.72%)	255 (55.19%)		
Q3, n (%)			0.251	< 0.001^1^	Q13, n (%)			0.2181	< 0.001
No	200 (42.46%)	89 (19.26%)			No	318 (67.52%)	212 (45.89%)		
Yes	271 (57.54%)	373 (80.74%)			Yes	153 (32.48%)	250 (54.11%)		
Q4, n (%)			0.167	< 0.001^1^	Q14, n (%)			0.2461	< 0.001
No	182 (38.64%)	107 (23.16%)			No	321 (68.15%)	202 (43.72%)		
Yes	289 (61.36%)	355 (76.84%)			Yes	150 (31.85%)	260 (56.28%)		
Q5, n (%)			0.205	< 0.001^1^	Q15, n (%)			0.1401	< 0.001
No	194 (41.19%)	102 (22.08%)			No	176 (37.37%)	113 (24.46%)		
Yes	277 (58.81%)	360 (77.92%)			Yes	295 (62.63%)	349 (75.54%)		
Q6, n (%)			0.25	< 0.001^1^	Q16, n (%)			0.2221	< 0.001
No	197 (41.83%)	87 (18.83%)			No	202 (42.89%)	102 (22.08%)		
Yes	274 (58.17%)	375 (81.17%)			Yes	269 (57.11%)	360 (77.92%)		
Q7, n (%)			0.258	< 0.001^1^	Q17, n (%)			0.1671	< 0.001
No	201 (42.68%)	87 (18.83%)			No	191 (40.55%)	115 (24.89%)		
Yes	270 (57.32%)	375 (81.17%)			Yes	280 (59.45%)	347 (75.11%)		
Q8, n (%)			0.189	< 0.001^1^	Q18, n (%)			0.0101	0.762
No	185 (39.28%)	101 (21.86%)			No	185 (39.28%)	177 (38.31%)		
Yes	286 (60.72%)	361 (78.14%)			Yes	286 (60.72%)	285 (61.69%)		
Q9, n (%)			0.195	< 0.001^1^	Q19, n (%)			0.0201	0.532
No	176 (37.37%)	91 (19.70%)			No	320 (67.94%)	305 (66.02%)		
Yes	295 (62.63%)	371 (80.30%)			Yes	151 (32.06%)	157 (33.98%)		
Q10, n (%)			0.194	< 0.001^1^	Q20, n (%)			0.0051	0.89
No	173 (36.73%)	89 (19.26%)			No	315 (66.88%)	307 (66.45%)		
Yes	298 (63.27%)	373 (80.74%)			Yes	156 (33.12%)	155 (33.55%)		

^1^Pearson’s Chi-squared test. All Q1–Q17 had uncorrected *p* < 0.001, which remain < 0.0025 after correction. Q18–Q20 were non-significant before and after correction.

“With the exception of items Q18 to Q20, all other items showed statistically significant differences in the proportion of correct responses between the two groups, with a significantly higher number of correct responses in the AR group (all *p* < 0.001 after Bonferroni correction). The effect sizes (Cramér’s V) for these 17 items ranged from 0.123 to 0.265, indicating small-to-medium associations with AR status. For the three non-significant items (Q18–Q20), the effect sizes were negligible (Cramér’s V ≤ 0.020), confirming no meaningful difference between groups.”

Table 3 presents the survey results regarding knowledge and attitudes. The analysis revealed significant distribution differences between the two groups for most survey items: a greater proportion of AR participants expressed a desire for the development of a curative medication, whereas a higher percentage of non-AR participants prioritized identifying the underlying causes of AR. Furthermore, most non-AR participants reported difficulty empathizing with the suffering experienced by AR patients, with this difference between groups being statistically significant. Notably, both groups exhibited a predominantly negative attitude toward the potential curability of AR, with over 50% of participants expressing disbelief in its cure. In terms of practice, participants in the AR group demonstrated a significantly stronger intention to adopt preventive or management behaviors (*p* < 0.05).

**TABLE 3:** The distribution of attitudes and practices between the AR group and the Non-AR group.

Characteristic	Allergic rhinitis		Non-Allergic rhinitis		
	*N* = 462		*N* = 471		
	Agree	Disagree	Agree	Disagree	*p*-value
Attitude					
Allergic rhinitis can be cured.	163 (35.28%)	299 (64.72%)	155 (32.91%)	316 (67.09%)	0.445
Understand or imagine the pain of patients with rhinitis.	351 (75.97%)	111 (24.03%)	171 (36.31%)	300 (63.69%)	** < 0.001**
Understand the causes of allergic rhinitis.	322 (69.70%)	140 (30.30%)	369 (78.34%)	102 (21.66%)	**0.003**
Research drugs for curing allergic rhinitis.	369 (79.87%)	93 (20.13%)	336 (71.34)	135 (28.66%)	**0.002**
Practice					
Prevent allergic rhinitis through relevant knowledge.	224 (48.48%)	238 (51.52%)	175 (37.15%)	296 (62.85%)	** < 0.001**
Wear masks to reduce the occurrence of rhinitis.	333 (72.08%)	129 (27.92%)	149 (31.63%)	322 (68.37%)	**< 0.001**

The results show a statistically significant difference. *p* < 0.05.

To investigate factors influencing knowledge scores, a cut-off point of ≥ 12 was defined in this study, with participants scoring ≥ 12 classified as the “relatively knowledgeable” group and those scoring < 12 as the “relatively knowledge-deficient” group. A logistic regression model was constructed to analyze variables potentially associated with knowledge scores. As shown in Table 4, the results indicated that having AR and being female were significantly associated with higher knowledge scores, suggesting that participants with AR and female participants possessed a better understanding of AR-related knowledge. In contrast, no significant correlations were observed between knowledge scores and other factors such as age, education level, marital status, or family history of AR.

**TABLE 4:** logistic regression analysis based on the differences in basic variables and scores.

Basic variables		OR, 95% CI			
		OR	LOR	UOR	*P* value
AR	No	Ref.			
	Yes	3.78	2.88	4.96	**< 0.001**
Age		0.98	0.97	1	0.068
Education	Graduate student	Ref.			
	High school and below	0.52	0.19	1.39	0.192
	Undergraduate	0.7	0.4	1.21	0.202
Sex	Male	Ref.			
	Female	1.54	1.17	2.04	**0.002**
Marital status	Married	Ref.			
	Unmarried	0.75	0.51	1.1	0.136
Family with AR	Yes	Ref.			
	No	0.85	0.71	1.02	0.086
Home location	City	Ref.			
	Rural areas	0.94	0.71	1.24	0.659

The results show a statistically significant difference. *p* < 0.05.

To control for potential confounding effects of covariates on the association between AR status and knowledge scores, a second regression model was constructed (Table 5). In this model, gender, age, educational level, marital status, family history of AR, and geographical location were included as covariates, with AR status serving as the independent variable and knowledge deficiency (score < 12) as the dependent variable. The results indicated that AR status remained a significant independent factor associated with knowledge scores. Specifically, non-AR participants demonstrated poorer knowledge of allergy-related information compared to their AR counterparts.

**TABLE 5:** logistic regression analysis based on AR and score differences.

Basic variables		OR 95% CI			
		OR	LOR	UOR	*P* value
AR (Unadjusted)	No	Ref.			
	Yes	3.78	2.88	4.96	**< 0.001**
AR (Adjusted)	No	Ref.			
	Yes	3.69	2.8	4.85	**< 0.001**

Adjusted: Gender, age, educational level, marital status, family history of allergic rhinitis, and geographical location of residence. The results show a statistically significant difference. *p* < 0.05.

## Sensitivity analysis results

To test the robustness of the threshold for knowledge score classification, we used a new dividing point of 11 points for the total knowledge score (the original main analysis used 12 points as the dividing point) and conducted a logistic regression analysis again. As shown in Table 6, without adjusting for confounding factors, the OR value for AR patients was 3.08 (95% CI: 2.32–4.09, *P* < 0.001); after adjusting for gender, age, educational level, marital status, AR family history, and place of residence, the OR value was 3.06 (95% CI: 2.30–4.08, *P* < 0.001). This result is consistent with the main analysis (with 12 points as the dividing point, the adjusted OR = 3.69, 95% CI: 2.80–4.85) and still has statistical significance, indicating that the positive correlation between the AR status and knowledge level does not depend on the specific score classification threshold, and the research conclusion is relatively robust.

**TABLE 6:** Sensitivity analysis, using knowledge score 11 as the cutoff criterion.

Basic variables		OR 95% CI			
		OR	LOR	UOR	*P* value
AR (Unadjusted)	No	Ref.			
	Yes	3.08	2.32	4.09	**< 0.001**
AR (Adjusted)	No	Ref.			
	Yes	3.06	2.3	4.08	**< 0.001**

Adjusted: Gender, age, educational level, marital status, family history of allergic rhinitis, and geographical location of residence. The results show a statistically significant difference. *p* < 0.05.

## Discussion

This study constitutes a KAP investigation focusing on AR-related knowledge. Our findings demonstrate that AR status serves as a pivotal determinant influencing knowledge scores. A survey published in 2025 examining caregivers of children with allergies similarly reported that caregivers of allergic children attained higher knowledge scores (13). Congruent with our results, its regression analysis identified family history of AR as a potential factor contributing to score differentials (Non-AR vs AR, OR = 0.85 [95%CI 0.71–1.02], *p* = 0.086). Extending these observations, our analysis revealed that gender represents another critical factor, with female participants exhibiting a higher probability of achieving superior scores compared to males. No statistically significant influence on score variance was identified for the remaining examined variables, including age, educational attainment, marital status, or geographic location of residence.

An unexpected finding was the lack of a significant association between higher educational attainment and superior knowledge scores. This suggests that while AR is a prevalent condition in daily life, its related knowledge is not part of general common sense; proactive learning appears necessary to acquire a deeper understanding. Furthermore, the distribution of knowledge scores revealed several notable areas of weakness. Both groups demonstrated pronounced knowledge deficiencies in five specific domains: home renovation (14, 15), humidity control (16, 17), probiotic foods (18, 19), active or passive smoking exposure during pregnancy (20), and mode of delivery (21, 22). These findings indicate that future AR-related health education and science communication should closely follow the latest research developments and incorporate contemporary international findings more comprehensively, thereby enhancing both the timeliness and the professional rigor of AR-related public knowledge dissemination.

Notably, at the attitudinal level, a significant majority of AR patients (64.72%) expressed distrust in current treatments, coupled with a strong desire to understand the pathogenesis of AR and the development of therapeutic drugs. Our study indicates that increased knowledge did not translate into comprehensive optimism regarding the nature of the disease. This paradox of “high knowledge yet low optimism” highlights potential structural deficiencies in current AR health education. While educational content may emphasize pathological mechanisms and treatment options, it appears to fall short in effectively conveying scientific expectations concerning long-term disease management, control targets, and quality of life. This has led to a societal status where patients are “informed” but not “convinced. “Furthermore, the attitude dimension revealed deeper socio-psychological phenomena. The marked contrast between AR patients’ urgent expectation for a “curative drug” and the non-AR population’s exploratory interest in the “etiological causes” reflects a fundamental divergence in health priorities between “experiencers” and “observers.” Experiencers, driven by symptom burden, seek direct relief from suffering, whereas observers approach the topic from a perspective of cognitive curiosity, focusing more on disease origins. More critically, over half of the non-AR participants admitted they “could not imagine the suffering of AR patients,” exposing a significant social empathy deficit. This lack of empathy contributes to the societal downplaying of AR’s disease burden, often leading to its mischaracterization as a minor inconvenience rather than a chronic condition capable of significantly impairing quality of life. This social perception represents a key psychological root of the long-standing dilemma AR faces: “high prevalence coupled with low societal attention.”

On the practice level, AR patients demonstrated stronger intentions to adopt health-promoting behaviors, which partially aligns with the “knowledge-attitude-practice” driving logic of the KAP model. This willingness represents a valuable intrinsic resource for connecting cognition with behavioral change and achieving effective self-management. However, translating this willingness into long-term, standardized, and sustainable actions remains a critical challenge for clinical intervention. It also reminds that future patient support must move beyond merely providing knowledge, to also offering the tools, skills, and social support necessary for behavioral change, thereby bridging the gap between “intention” and “sustained action.”

## Limitations

This study has several limitations. First, its cross-sectional design precludes establishing causal relationships among KAP factors, limiting findings to associations. Future intervention studies should employ active control groups (e.g., standard health education) to distinguish intervention-specific effects. Second, limitations related to sampling and representativeness should be noted. The use of convenience sampling means the sample may not be fully representative of the broader AR and non-AR populations, introducing potential selection bias. Additionally, questionnaires from minors were excluded during the analysis stage, which might further affect representativeness—for example, if minors with more severe conditions were systematically excluded, this could bias the knowledge scores in the AR group. Moreover, as this was a web-based survey, individuals with limited access to or familiarity with electronic devices (e.g., elderly or less educated populations) were likely underrepresented, introducing an additional layer of internet-based selection bias. Third, KAP data relied on self-reporting, which may be subject to social desirability bias, creating discrepancies between reported responses and actual behaviors. Fourth, the data collection period (September to December 2022, from autumn to early winter in Chengdu) may have introduced seasonal effects. During this period, pollen concentrations decrease, but indoor allergens such as dust mites and mold may increase. These seasonal variations could influence symptom severity and healthcare-seeking behaviors among AR patients, thereby indirectly affecting their KAP scores. Fifth, the study did not further explore potential KAP differences among patients with different AR subtypes (e.g., perennial vs. seasonal) or varying severity levels.

Notwithstanding these limitations, the study’s strengths include a relatively large sample size, a comprehensive assessment of KAP components, and a rigorous, logical analytical process. The findings reflect certain aspects of the knowledge-attitude-practice status among AR and non-AR populations, providing valuable clinical insights for future AR prevention strategies.

## Conclusion

This study, through a comparative analysis of the knowledge, attitudes, and practice characteristics between participants with AR and non-AR controls, revealed systematic differences between the two groups in disease awareness, attitudinal tendencies, and behavioral intentions. The AR group demonstrated significantly higher knowledge scores compared to the non-AR group. However, several knowledge gaps persist and warrant further attention. Furthermore, AR patients exhibited a lack of confidence in current treatments, suggesting a need for greater focus on psychological guidance and support for this population in future clinical management strategies.

## Data Availability

The datasets presented in this study can be found in online repositories. The names of the repository/repositories and accession number(s) can be found in the article/Supplementary material.
