# Supplementary material for: Attitudes, and practices toward allergic rhinitis: a comparative cross-sectional study of patients and non-patients in China
Source: Front Med (Lausanne). 2026 Jun 8;13:1807065. doi: 10.3389/fmed.2026.1807065 (PMC13284856; doi:10.3389/fmed.2026.1807065)
Supplement: Supplementary file 1 [file Supplementary_file_1.docx]

**A survey on knowledge, attitude and practice of allergic rhinitis**

With environmental changes and lifestyle alterations, the incidence of allergic rhinitis has been continuously rising worldwide. Sneezing, runny nose, itchy nose, nasal congestion... These symptoms not only affect daily life but also may interfere with sleep, work and study. Unfortunately, many people still have insufficient understanding of allergic rhinitis and even mistake it for "common cold", resulting in delayed diagnosis and treatment.

In order to gain a more comprehensive understanding of the public's knowledge level, attitude tendencies, and daily prevention behaviors regarding allergic rhinitis, we have specially conducted this questionnaire survey. Your genuine responses will help us identify common knowledge misconceptions and provide a scientific basis for conducting more targeted health education in the future.

If you agree to participate in this survey, please fill in according to your actual situation. If you do not agree, you can give up filling in. (Our team assumes that all questionnaire fillers have informed consent to this research, and promises that the results of this survey will only be used for student psychological research)

**Basic character information：**

1.What's your gender, please？

A. Male B. Female

2.How old are you？

3.Your height

cm

4.Your weight

Kg

5.Your education

High school and below Undergraduate Graduate student

6.Your Marital status

Married Unmarried

7.Your home location.

City Rural areas

8.Do you have a family history of allergies?

Yes No

**Knowledge**

K1 Do you know that frequent exposure to pollen is more likely to cause allergic rhinitis ?

Yes No

K2 Do you know that active or passive smoking is more likely to cause allergic rhinitis ?

Yes No

K3 Do you know that long-term inhalation of irritating odor is more likely to cause allergic rhinitis ?

Yes No

K4 Do you know that long-term living in areas with heavy air pollution is more likely to develop allergic rhinitis ?

Yes No

K5 Do you know that when the external temperature is high, the symptoms of rhinitis will be aggravated and allergic rhinitis will be more likely to occur ?

Yes No

K6 Do you know that sudden exposure to cold air can cause nasal symptoms in some patients with allergic rhinitis ?

Yes No

K7 Do you know that patients with allergic rhinitis are more likely to attack after thunderstorm weather ?

Yes No

K8 Do you know that long-term use of antibiotics will be more likely to suffer from allergic rhinitis ?

Yes No

K9 Do you know that children whose parents have allergic rhinitis or other allergic diseases are more likely to suffer from allergic rhinitis ?

Yes No

K10 Do you know that people with food allergies are more likely to suffer from allergic rhinitis ?

Yes No

K11 Do you know that living in a room that is not cleaned for a long time will be more prone to allergic rhinitis ?

Yes No

K12 Do you know that living in a newly renovated house will be more prone to allergic rhinitis ?

Yes No

K13 Do you know that long-term living in a high humidity environment is more prone to allergic rhinitis ?

Yes No

K14 Do you know that active consumption of probiotic foods ( yogurt, pickles, vinegar, etc. ) can reduce the incidence of allergic rhinitis ?

Yes No

K15 Do you know that eating high fiber-rich foods ( fruits, vegetables, whole grains, beans, etc. ) can reduce the incidence of allergic rhinitis ?

Yes No

K16 Do you know that overweight is more likely to suffer from allergic rhinitis ?

Yes No

K17 Do you know that frequent exposure to dust mites is prone to nasal symptoms or even allergic rhinitis ?

Yes No

K18 Do you know that exposure to long-term unwashed plush items can aggravate nasal symptoms ?

Yes No

K19 Do you know that new children are prone to allergic rhinitis after active or passive smoking before and during pregnancy ?

Yes No

K20 Do you know that children born through cesarean section are more likely to suffer from allergic rhinitis ?

Yes No

Attitude

A1 I hope to understand the causes of allergic rhinitis.

A2 I hope to research drugs for curing allergic rhinitis.

A3 I can understand or imagine the pain of patients with rhinitis.

A4 I believe that allergic rhinitis be cured.

Practice

P1 I will prevent allergic rhinitis through relevant knowledge.

Yes No

P2 I will reduce the onset of rhinitis by wearing a mask.

Yes No
