# Supplementary material for: Attitudes, and practices toward allergic rhinitis: a comparative cross-sectional study of patients and non-patients in China
Source: Front Med (Lausanne). 2026 Jun 8;13:1807065. doi: 10.3389/fmed.2026.1807065 (PMC13284856; doi:10.3389/fmed.2026.1807065)
Supplement: Supplementary file 2 [file Table_1.docx]

sTable1: Analysis of the expert consultation results for the questionnaire items

| Item | Expert1 | Expert2 | Expert3 | Expert4 | Expert5 | Expert6 | Mean | SD | CV | Interpretation |
| --- | --- | --- | --- | --- | --- | --- | --- | --- | --- | --- |
| Q1 | 5 | 4 | 4 | 5 | 5 | 5 | 4.67 | 0.52 | 0.11 | High importance, high consistency |
| Q2 | 3 | 3 | 4 | 4 | 3 | 3 | 3.33 | 0.52 | 0.16 | Moderate importance, good consistency |
| Q3 | 3 | 3 | 4 | 4 | 4 | 3 | 3.5 | 0.55 | 0.16 | Moderate importance, good consistency |
| Q4 | 4 | 4 | 3 | 3 | 4 | 5 | 3.83 | 0.75 | 0.2 | Good importance, acceptable consistency |
| Q5 | 4 | 5 | 4 | 5 | 3 | 5 | 4.33 | 0.82 | 0.19 | High importance, good consistency |
| Q6 | 2 | 2 | 3 | 2 | 3 | 3 | 2.5 | 0.55 | 0.22 | Low-moderate importance, acceptable consistency |
| Q7 | 2 | 2 | 3 | 1 | 2 | 4 | 2.33 | 0.98 | 0.42 | Low importance, large disagreement |
| Q8 | 5 | 5 | 4 | 5 | 4 | 5 | 4.67 | 0.52 | 0.11 | High importance, high consistency |
| Q9 | 5 | 2 | 4 | 3 | 3 | 5 | 3.67 | 1.21 | 0.33 | Moderate-high importance, moderate disagreement |
| Q10 | 5 | 4 | 4 | 4 | 4 | 5 | 4.33 | 0.52 | 0.12 | High importance, high consistency |
| Q11 | 5 | 4 | 4 | 5 | 3 | 5 | 4.33 | 0.82 | 0.19 | High importance, good consistency |
| Q12 | 3 | 3 | 3 | 2 | 3 | 4 | 3 | 0.63 | 0.21 | Moderate importance, good consistency |
| Q13 | 2 | 2 | 3 | 1 | 2 | 2 | 2 | 0.63 | 0.32 | Low importance, acceptable consistency |
| Q14 | 3 | 3 | 3 | 3 | 2 | 3 | 2.83 | 0.41 | 0.14 | Moderate importance, high consistency |
| Q15 | 4 | 4 | 4 | 4 | 4 | 5 | 4.17 | 0.41 | 0.1 | High importance, high consistency |
| Q16 | 5 | 5 | 4 | 5 | 5 | 5 | 4.83 | 0.41 | 0.08 | Very high importance, excellent consistency |
| Q17 | 5 | 3 | 4 | 5 | 3 | 5 | 4.17 | 0.98 | 0.24 | High importance, acceptable consistency |
| Q18 | 5 | 4 | 4 | 4 | 4 | 5 | 4.33 | 0.52 | 0.12 | High importance, high consistency |
| Q19 | 3 | 3 | 3 | 1 | 3 | 3 | 2.67 | 0.82 | 0.31 | Low-moderate importance, acceptable consistency |
| Q20 | 3 | 4 | 3 | 1 | 3 | 3 | 2.83 | 0.98 | 0.35 | Low importance, large disagreement (borderline) |
| A1 | 5 | 4 | 5 | 5 | 4 | 5 | 4.67 | 0.52 | 0.11 | High importance, high consistency |
| A2 | 5 | 5 | 5 | 5 | 5 | 5 | 5 | 0 | 0 | Perfect consistency, extremely high importance |
| A3 | 4 | 4 | 4 | 4 | 3 | 5 | 4 | 0.63 | 0.16 | High importance, good consistency |
| A4 | 4 | 4 | 5 | 3 | 4 | 5 | 4.17 | 0.75 | 0.18 | High importance, good consistency |
| P1 | 4 | 2 | 5 | 2 | 2 | 4 | 3.17 | 1.17 | 0.37 | Moderate importance, large disagreement |
| P2 | 4 | 3 | 3 | 4 | 2 | 3 | 3.17 | 0.75 | 0.24 | Moderate importance, acceptable consistency |
